# A cardiotoxicity dataset for breast cancer patients

**DOI:** 10.1038/s41597-023-02419-1

**Published:** 2023-08-08

**Authors:** Beatriz Piñeiro-Lamas, Ana López-Cheda, Ricardo Cao, Laura Ramos-Alonso, Gabriel González-Barbeito, Cayetana Barbeito-Caamaño, Alberto Bouzas-Mosquera

**Affiliations:** 1 Grupo MODES, Departamento de Matemáticas, CITIC, Universidade da Coruña, A Coruña, 15071 Spain; 2 Servicio de Medicina Interna, Complexo Hospitalario Universitario de A Coruña, A Coruña, 15006 Spain; 3 Unidad de Imagen y Función Cardíaca, Servicio de Cardiología, Complexo Hospitalario Universitario de A Coruña, Instituto de Investigación Biomédica de A Coruña (INIBIC), A Coruña, 15006 Spain

**Keywords:** Image processing, Breast cancer

## Abstract

This dataset is a result of the collaboration between the University of A Coruña and the University Hospital of A Coruña. It contains information about 531 women diagnosed with HER2+ breast cancer, treated with potentially cardiotoxic oncologic therapies. These treatments can cause cardiovascular adverse events, including cardiac systolic dysfunction, the development of which has important clinical and prognostic implications. The availability of good predictors may enable early detection of these cardiac problems. Variables such as age, weight and height are available for each patient, as well as some measures obtained from echocardiography, a technique used prior and during the treatment to check the structure and function of the heart. Among them, there is a functional variable that measures the myocardial velocity during the cardiac cycle. For patients that experienced cancer therapy-related cardiac dysfunction during the treatment period, time until its appearance is known. This dataset aims to enable the scientific community in conducting new research on this cardiovascular side effect.

## Background & Summary

**Fig. 1:**
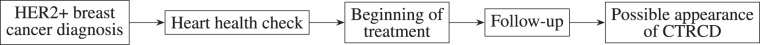
Schematic overview of the treatment process.

Breast cancer is the most common cancer in women. According to the World Health Organization, 2.3 million of women were diagnosed with breast cancer around the world in 2020, and 685000 died due to this cause^1^. Around 20% of all breast cancers have higher levels of the protein human epidermal growth factor receptor 2 (HER2), that helps breast cancer cells grow quickly. They are known as HER2-positive (HER2+). These cancers tend to grow and spread faster than breast cancers that are HER2-negative (HER2-), but are much more likely to respond to treatment with drugs that target the HER2 protein. Previous studies have confirmed that therapies that specifically target HER2 (for example, trastuzumab) have a strong antitumor effect, improving the overall survival and the progression-free survival^2^. However, anti-HER2 therapies may cause cardiotoxicity^3^. In addition, they can be combined with other antineoplastic drugs that may also have cardiotoxic effects. Figure 1 shows a flowchart of the treatment process. Baseline clinical and image variables are obtained during the checkup. The development of cancer therapy-related cardiac dysfunction (CTRCD) has important prognostic implications, so its early detection is an ongoing challenge. Previous studies found that clinical factors such as age, hypertension, previous anthracycline treatment or low baseline left ventricular ejection fraction (LVEF), among others, are risk factors^4^, but they are not sufficient to predict which patients will develop CTRCD during treatment. Therefore, in clinical practice it is recommended to monitor the appearance of CTRCD through blood laboratory tests and cardiac imaging tests. The echocardiogram is the fundamental test, since it allows to assess various parameters of the structure and function of the heart, such as LVEF. This parameter gives the percentage of blood leaving the heart each time it contracts. CTRCD is detected when the LVEF falls below 50 and by at least 10 points from the baseline LVEF^5^. In addition, the Tissue Doppler Imaging (TDI), which shows the velocity of contraction and relaxation of the cardiac muscle, can be obtained by using these techniques. This information, measured before treatment, may help in the early identification of CTRCD. Since the TDI shows a velocity as a function of time, it can be preprocessed to obtain a functional datum. However, the analysis of image and functional covariates as risk factors has not been broadly examined in the existing literature. This is one of the reasons that motivated building this dataset. Furthermore, in the study of cardiotoxicity risk factors, time until its appearance is often ignored. The availability of the aforementioned time in this dataset opens the opportunity to use survival analysis techniques in this context. To the best of our knowledge, there are no other accessible TDI datasets in the literature containing a time to cardiotoxicity variable. VigiBase^6^ is the unique WHO global dataset of reported potential side effects of medicinal products. It includes over 30 million reports of suspected adverse effects of medicines, and it is continuously updated with incoming reports. However, a fee may be charged to access the data. Regarding other datasets available in this field, Venturelli *et al*.^7^ evaluated the efficacy of Tissue Doppler Imaging in the early detection of myocardial alterations in childhood cancer survivors. However, they did not consider the TDI image itself, but instead some parameters obtained from it. In addition, time until the appearance of the cardiac problem was not measured. Suran *et al*.^8,9^ evaluated the role of TDI in the assessment of global left and right ventricular function in type 1 diabetes mellitus patients. Neither TDI images nor time until the appearance of the cardiac problem were included in the dataset. Moreover, the cardiac problem is not an adverse effect of the treatment (but of the diabetes itself). We compared the characteristics of the above-mentioned datasets and the one proposed in this paper^10^ (see Table 1).

**Table 1:** Cardiac side effects dataset comparison.

Authors	*n*	Clinical variables	Functional or image variables	Time until cardiac problem	Treatment	Dose
Piñeiro-Lamas *et al*.^10^	531	Yes	Yes	Yes	anti-HER2 anthracyclines	No
Venturelli *et al*.^7^	50	Yes	No	No	anthracyclines	Yes
Suran *et al*.^8,9^	53	Yes	No	No	insulin	No

## Methods

### Participants

The authors declare that this study was conducted in accordance with the principles of the World Medical Association Declaration of Helsinki “Ethical Principles for Medical Research Involving Human Subjects”. The ethical approval for this research was obtained from the Drug Research Ethical Committee of Galicia (CEIm-G), code 2022/492. The IRB waived consent for the work as retrospective study.

Our data consists of information about 531 women diagnosed with HER2 + breast cancer between 2007 and 2021 and treated with potentially cardiotoxic oncologic drugs at the University Hospital of A Coruña. Among those patients, 54 (10.17 %) experienced CTRCD during the follow-up period. The age group with the highest prevalence (17.29 %) is 60–69. Table 2 shows the list of available variables, along with the acronym description (if needed), their type (numerical, binary or image) and their measure unit. All variables were measured prior to treatment. A detailed description of the participants’ characteristics is presented in Table 3 (numerical variables) and Table 4 (binary variables). The variable time contains, for patients who experienced CTRCD, the time (in days) until its appearance. For patients who did not experience this side effect, time contains the length of the follow-up period.

**Table 2:** Acronym description and basic information of the variables.

Acronym	Description (if needed)	Type	Unit of measure
**age**	—	numerical	years
**weight**	—	numerical	kg
**height**	—	numerical	cm
**CTRCD**	CTRCD experienced during the follow-up period	binary	0 (no) / 1 (yes)
**time**	time from beginning of treatment to CTRCD or end of follow-up	numerical	days
**LVEF**	Left Ventricular Ejection Fraction	numerical	%
**heart rate**	—	numerical	beats per minute (bpm)
**heart rhythm**	—	binary	0 (sinus rhythm) 1 (atrial fibrillation)
**PWT**	Posterior Wall Thickness	numerical	cm
**LAd**	Left Atrial diameter	numerical	cm
**LVDd**	Left Ventricular Diastolic diameter	numerical	cm
**LVSd**	Left Ventricular Systolic diameter	numerical	cm
**AC**	anthracyclines	binary	0 (no) / 1 (yes)
**antiHER2**	anti-HER2 therapies	binary	0 (no) / 1 (yes)
**ACprev**	previous anthracyclines	binary	0 (no) / 1 (yes)
**antiHER2prev**	previous anti-HER2 therapies	binary	0 (no) / 1 (yes)
**HTA**	hypertension	binary	0 (no) / 1 (yes)
**DL**	dyslipidemia	binary	0 (no) / 1 (yes)
**DM**	diabetes mellitus	binary	0 (no) / 1 (yes)
**smoker**	—	binary	0 (no) / 1 (yes)
**exsmoker**	—	binary	0 (no) / 1 (yes)
**RTprev**	previous thorax radiotherapy	binary	0 (no) / 1 (yes)
**CIprev**	previous cardiac insufficiency	binary	0 (no) / 1 (yes)
**ICMprev**	previous ischemic cardiomyopathy	binary	0 (no) / 1 (yes)
**ARRprev**	previous arrhythmia	binary	0 (no) / 1 (yes)
**VALVprev**	previous valvulopathy	binary	0 (no) / 1 (yes)
**valvsurgprev**	previous valve surgery	binary	0 (no) / 1 (yes)
**TDI**	Tissue Doppler Imaging	image	cm/s (once preprocessed)

**Table 3:** Baseline clinical numerical characteristics of participants.

Variable	Min	Q1	Median	Mean	Q3	Max
age	26	45.5	55	55.05	65	86
weight	40	58	66	68.3	75	120
height	138	155	159.5	159.3	164	186
time	5	278	518	855	1146	4276
LVEF	36.81	61.02	65.37	65.28	70.31	78.87
heart rate	46	65	73	74.61	82	138
PWT	0.4631	0.7638	0.8763	0.8757	0.9761	1.7100
LAd	1.662	3.000	3.317	3.325	3.636	5.000
LVDd	2.831	4.020	4.345	4.352	4.692	5.970
LVSd	1.258	2.459	2.746	2.765	3.000	4.700

**Table 4:** Baseline clinical binary characteristics of participants.

	*n*(%)	*n*(%)
**Variable**	**0**	**1**
**CTRCD**	477 (89.83)	54 (10.17)
**heart rhythm**	521 (98.67)	7 (1.33)
**AC**	150 (31.32)	329 (68.68)
**antiHER2**	139 (28.84)	343 (71.16)
**ACprev**	423 (87.76)	59 (12.24)
**antiHER2prev**	449 (93.15)	33 (6.85)
**HTA**	381 (79.21)	100 (20.79)
**DL**	390 (80.91)	92 (19.09)
**DM**	455 (94.4)	27 (5.6)
**smoker**	412 (86.01)	67 (13.99)
**exsmoker**	411 (85.98)	67 (14.02)
**RTprev**	415 (86.28)	66 (13.72)
**CIprev**	480 (99.59)	2 (0.41)
**ICMprev**	475 (98.55)	7 (1.45)
**ARRprev**	469 (97.3)	13 (2.7)
**VALVprev**	475 (98.55)	7 (1.45)
**cxvalv**	481 (99.79)	1 (0.21)

**Fig. 2:**
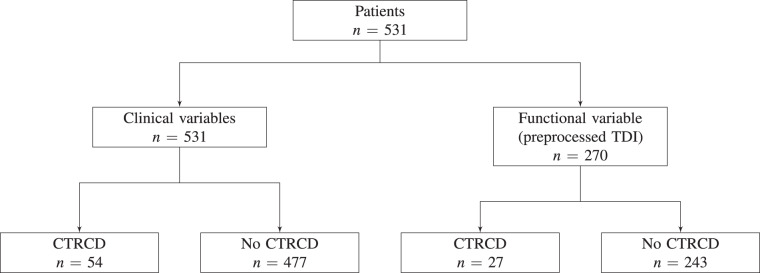
Schematic overview of the number of observations depending on the type of variable (clinical or functional) and the CTRCD status.

Regarding TDI, since it shows a velocity, it should be considered as a function, in spite of its image nature. A preprocessing was proposed and implemented in the statistical software R^11^ to obtain a functional covariate from the image. Although 531 images are available, only 270 were preprocessed. The reason is that the TDIs were obtained with different echocardiographic devices. For images provided by the most used device, a semi-automatic algorithm was designed to preprocess them and obtain the corresponding functions. For images provided by the rest of machines, automation was not achieved. Therefore, they were not preprocessed because they required excessive manual work. Figure 2 shows an overview of the number of available data based on the nature of the variable (clinical or functional) and the CTRCD status of the patient. A detailed description of the preprocessing procedure is presented in the following subsection.

### Preprocessing of image data

**Fig. 3:**
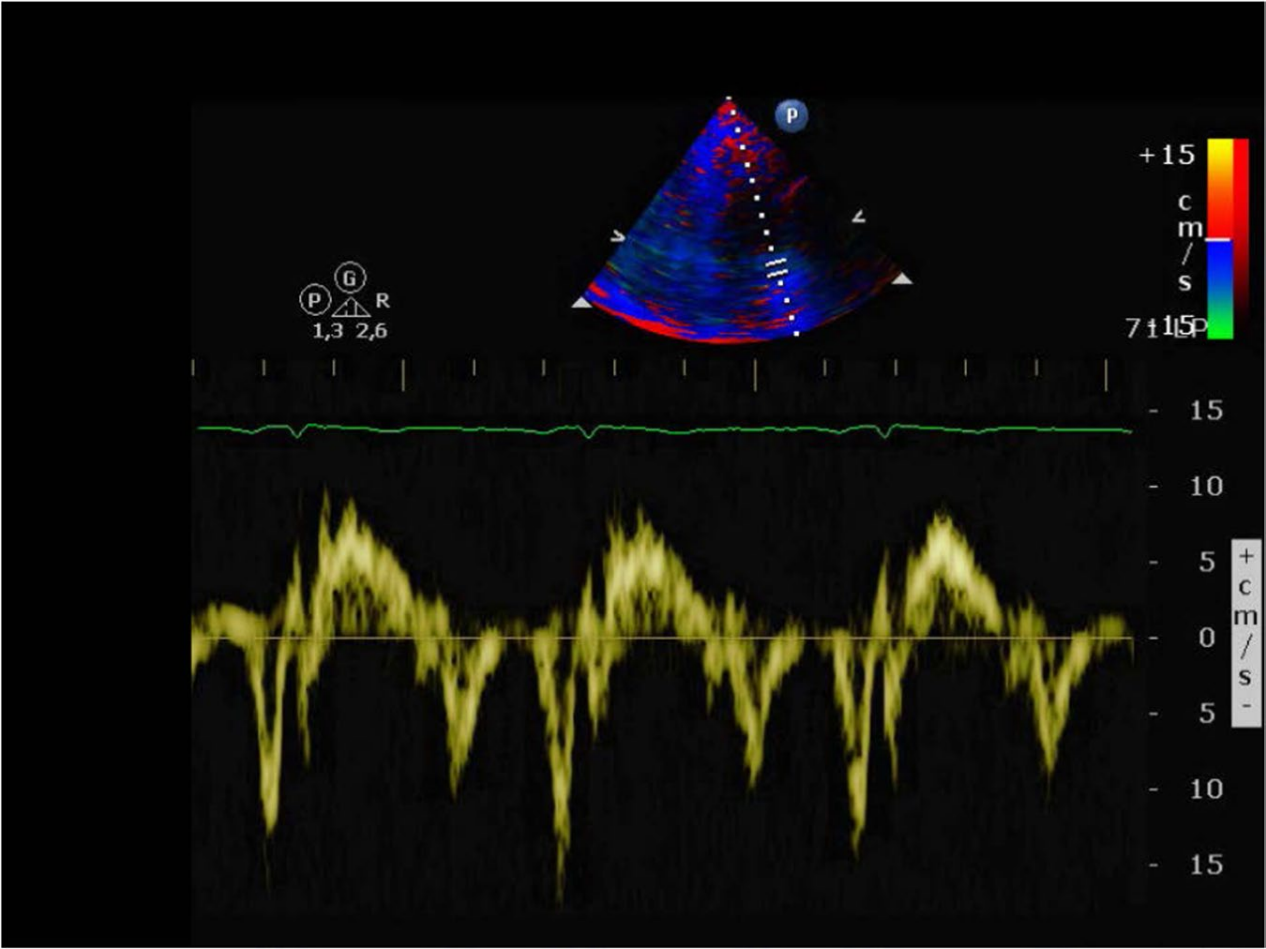
Example of a TDI.

**Fig. 4:**
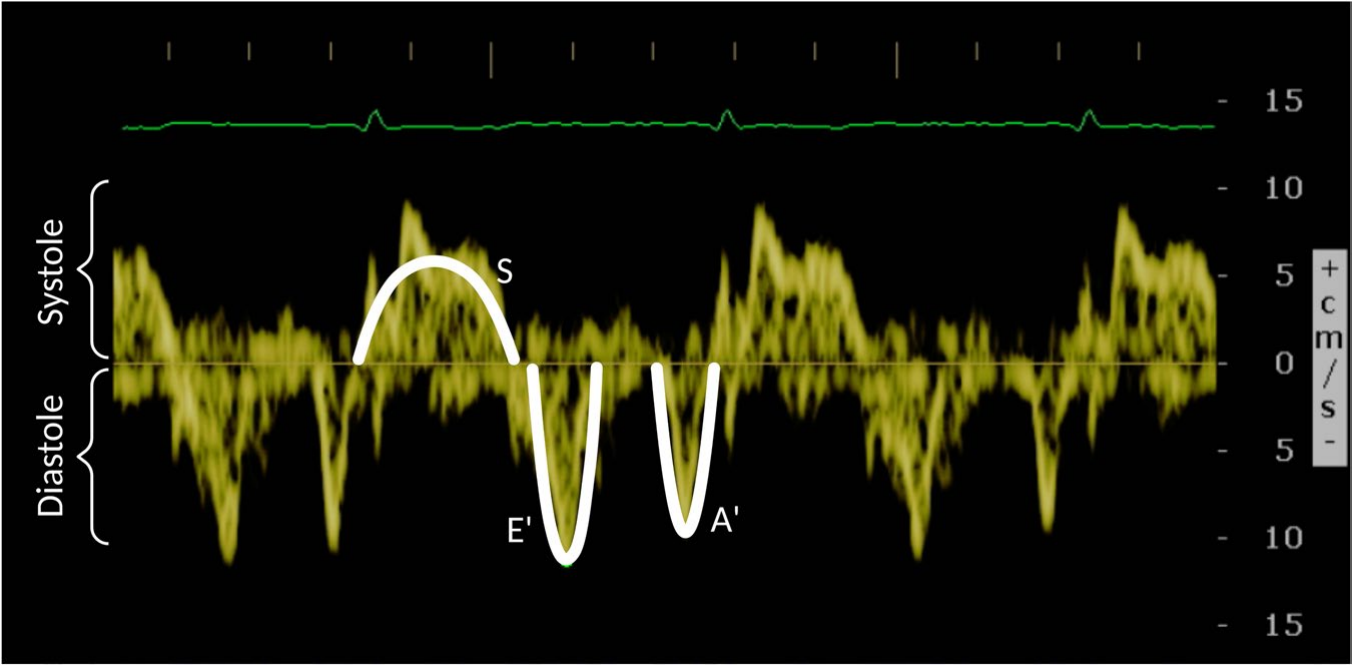
Illustration of S, E’ and A’ waves in a TDI.

**Fig. 5:**
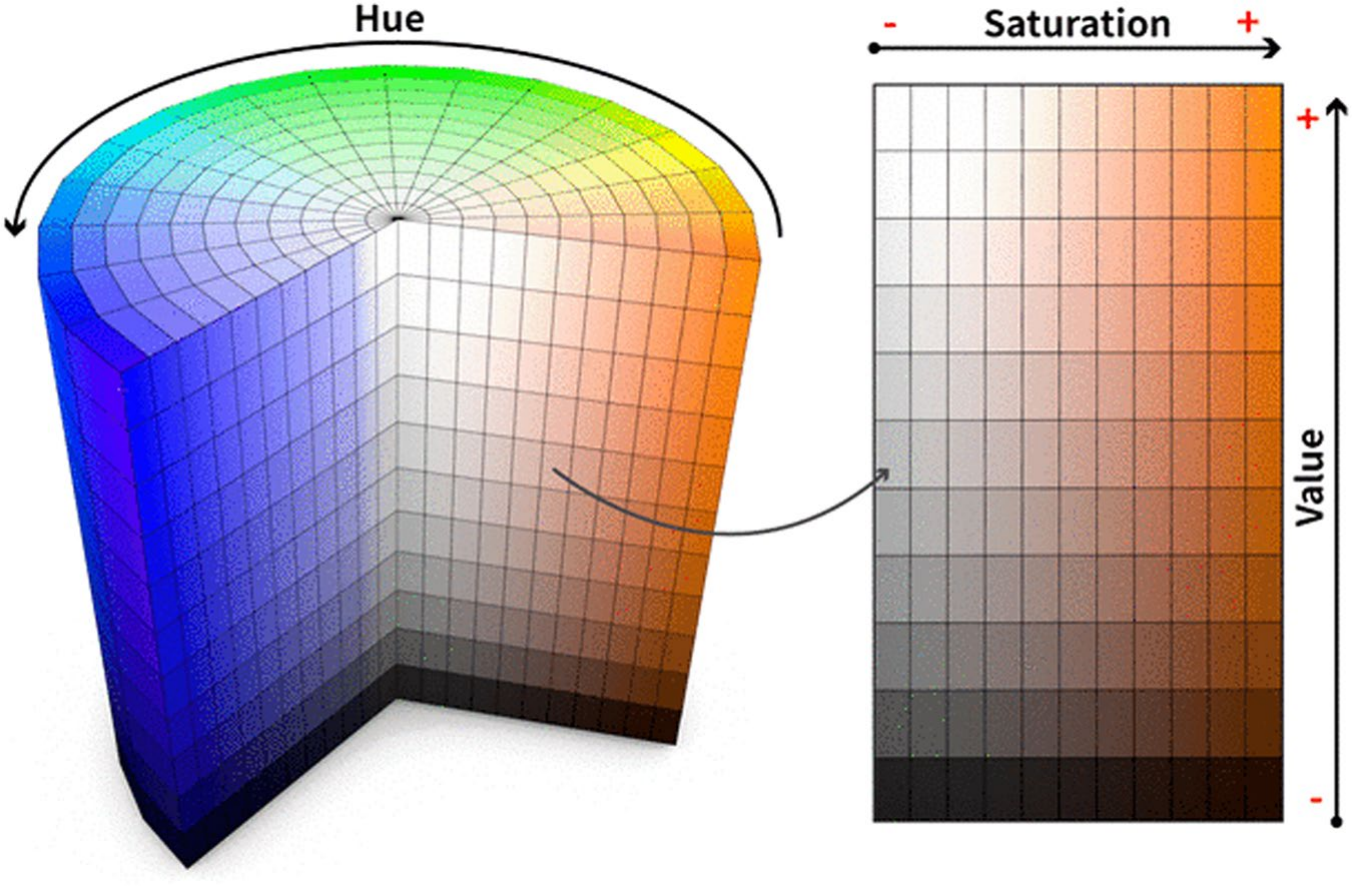
HSV colour model (cylinder type). Retrieved April 3, 2023, from Kim^18^.

The baseline TDI, which informs about the condition of the heart just before the treatment begins, may be a good predictor of CTRCD^12^. Figure 3 shows an example of a TDI. Although it is stored as an image, the main interest relies on the yellow signal, which shows the velocity of contraction and relaxation of the cardiac muscle. Note that the cardiac cycle is represented by 3 waveforms, as illustrated in Fig. 4: S (systolic), above zero velocity; and E’ and A’ (early and late diastolic), with negative velocity. In order to extract the underlying function, a preprocessing of the image was proposed by taking into account the colour of each pixel in HSV format (Fig. 5). Note that HSV stands for Hue-Saturation-Value:

- Hue ranges from 0 to 360 degrees, and it is the attribute of a colour by virtue of which it is discernible as red, green, yellow, etc. It depends on the dominant wavelength, but not on the intensity or lightness.
- Saturation describes the amount of gray in a particular colour, ranging from 0 (gray) to 1 (primary colour).
- Value describes the brightness or intensity, from 0 to 1, where 0 is completely black, and 1 is the brightest.

The preprocessing procedure consists of the following steps:

#### Step 1. Detection of the yellow pixels

The signal of interest has different yellow tones. Unlike the well-known colour plane representation red, green and blue (RGB), HSV is closer to how humans perceive colours. In HSV, the pixels in yellow can be identified just by controlling the range of movement of the first parameter (Hue). However, with RGB it is not that easy, since very different combinations of the three components may result in similar final colours. Therefore, it is challenging to choose a proper range for the three primary colours so that their mixture is yellow.

#### Step 2. Transformation into a function

**Fig. 6:**
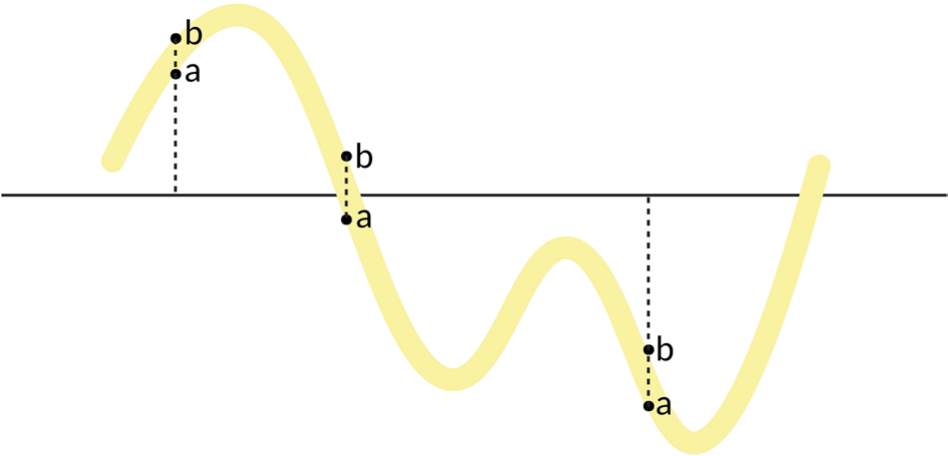
Illustration of *a* and *b* points in a TDI.

Once the yellow pixels have been detected, the result is still not a function, because for each value in the horizontal axis there is more than one yellow pixel in vertical. To overcome this drawback, the following procedure has been considered. For each pixel in the horizontal axis, let us define by *a* the lowest yellow pixel in vertical and by *b* the highest one (Fig. 6). Then, there will be three situations:

- If 0 < *a*  < *b*, *b* is considered.
- If *a* < *b* < 0, *a* is considered.
- If *a* < 0 < *b*, consider $a\frac{{e}^{-\lambda a/\left(b-a\right)}}{{e}^{-\lambda a/\left(b-a\right)}+{e}^{\lambda b/\left(b-a\right)}}+b\frac{{e}^{\lambda b/\left(b-a\right)}}{{e}^{-\lambda a/\left(b-a\right)}+{e}^{\lambda b/\left(b-a\right)}}$, for some *λ* > 0.

Preliminary studies (supervised by cardiology experts) suggested that a good choice for *λ* is 10. The result is a collection of pixel position pairs. To obtain their coordinates (time on the horizontal axis, velocity on the vertical one), we have taken into account the known coordinates of some pixels in the image. This way, a function for each patient can be obtained from the TDI image.

#### Step 3. Selection of one cycle per patient

The resulting function is a periodic combination of S, E’ and A’ waves, as illustrated in Fig. 4. The end of the A’ wave determines the end of a cycle and the beginning of the subsequent one. To standardize the data, just one cycle for each patient is selected considering the following criterion:

- If a patient’s function only contains one full cycle, this one is selected.
- If it has two cycles, the one that describes better the S, E’ and A’ waveforms (supervised by experts) is chosen.
- If it has three or more, the selected one is the one verifying that its distance to the mean cycle is the lowest.

**Fig. 7:**
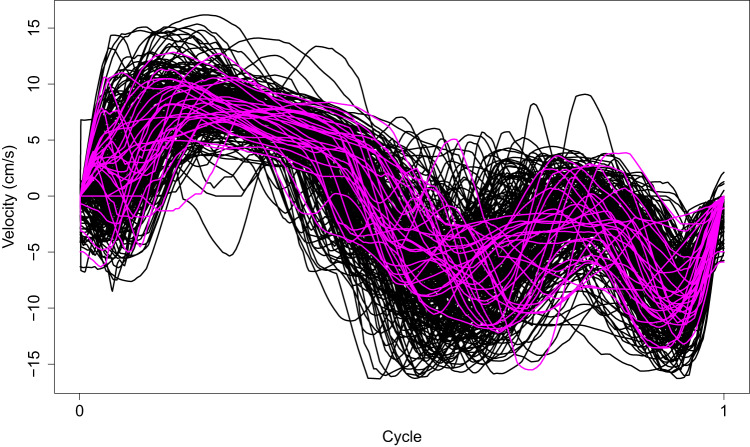
In magenta, the cycles of the patients who experienced CTRCD during the follow-up period. In black, the cycles of the patients who did not experience this side effect.

All the cycles have been standardized to have the same length (one cycle). Figure 7 shows the standardized cycles for the 270 patients. The difference between the cycles of both groups of patients (with and without CTRCD) is more remarkable in the central zone. In particular, for the 27 patients with CTRCD detected during the follow-up period, E’ and A’ waves seem smoother, as well as slightly shifted to the right.

## Data Records

Data presented in this work consist of three files

- BC_cardiotox_clinical_variables.csv: dataset with 531 rows (patients) and 27 columns (clinical variables in Table 2). The column headers are age, weight, height, CTRCD, time, LVEF, heart_rate, heart_rhythm, PWT, LAd, LVDd, LVSd, AC, antiHER2, ACprev, antiHER2prev, HTA, DL, DM, smoker, exsmoker, RTprev, CIprev, ICMprev, ARRprev, VALVprev and cxvalv.
- BC_cardiotox_functional_variable.csv: dataset with 270 rows (patients) and 1002 columns. The first column is CTRCD, and the remaining 1001 (named t 1, t 2, t 1000, t 1001) contain the cycle extracted from the TDI discretized in 1001 equispaced points in the interval [0,1].
- BC_cardiotox_clinical_and_functional_variables.csv: dataset with 531 rows (patients) and 1028 columns. The first 27 columns are the same as in BC_cardiotox_clinical_variables, and the remaining are t 1, t2, t 1000, t 1001. For the patients whose image has not been preprocessed, the last 1001 columns contain NAs.

These files are freely available online at Figshare^10^, as well as the 270 TDI images that were preprocessed. A README file with a detailed description of each dataset is also available.

## Technical Validation

**Fig. 8:**
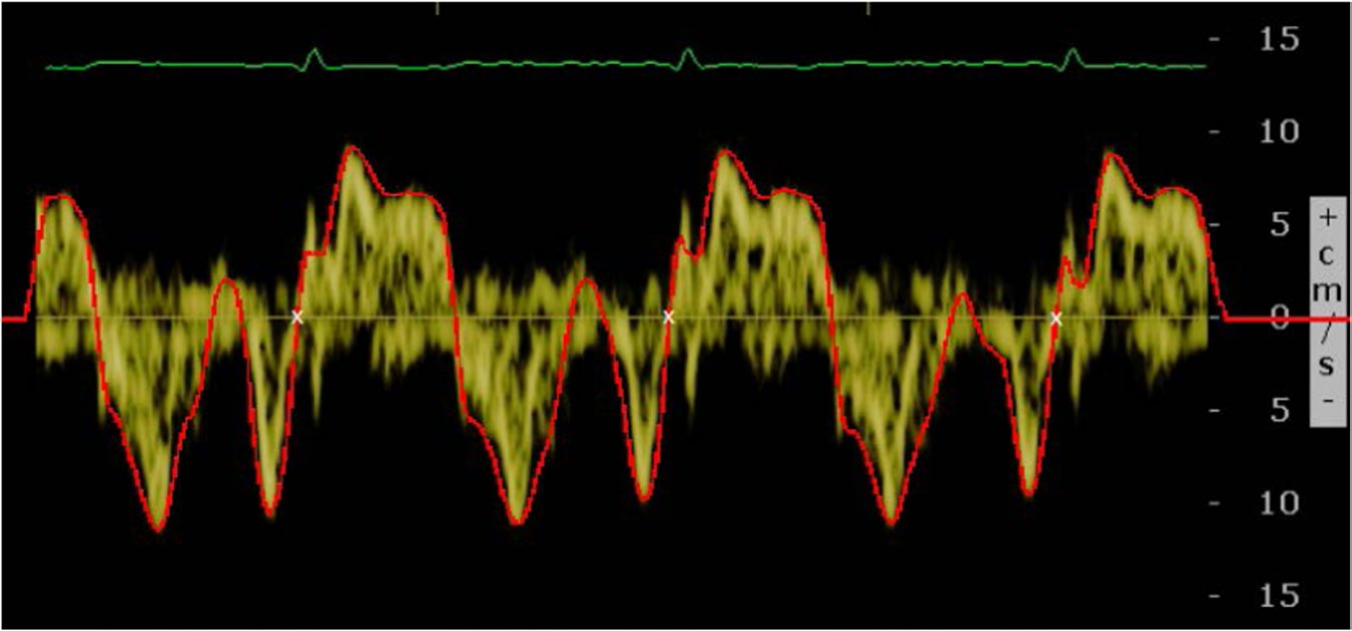
TDI image with its corresponding function (in red) and the beginning and end points of the cycles (with white crosses).

**Fig. 9:**
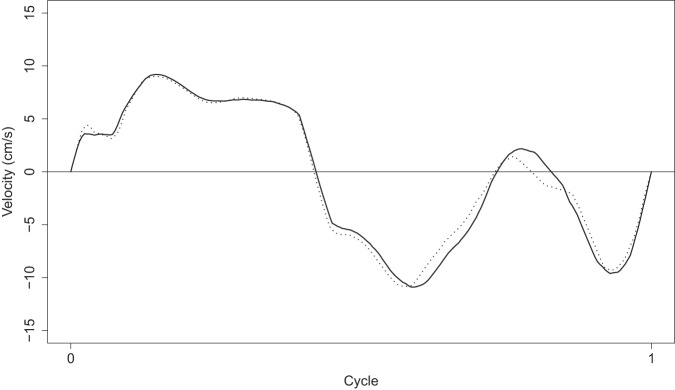
Cycles extracted from the previous image (the first one in solid line, the second one in dotted line), after being standardized to have the same length.

Preliminary exploratory analyses have made it possible to detect (and correct) some outliers and fill in some apparently missing data. Regarding the TDI images, the corresponding functions obtained with the preprocessing procedure described before have been examined one by one. The algorithm worked automatically for 137 of the 270 images. However, for the remaining 133, the selection of the cycle had to be manually done. In general, the selection of the cycle can not be automated when the function crosses the horizontal axis several times in the gap between A’ and S waves. This gap, known as isovolumetric contraction time (IVCT), is the first phase of systole. When the function intersects the axis during the IVCT more than once, the beginning and end of the cycles have to be manually selected from the set of intersection points. Moreover, there are situations where the cycle does not begin at zero velocity, and therefore the intersection points with the axis are not useful. In these cases, the beginning and end of the cycles have to be manually chosen too. For the automatically preprocessed images, 77% of the outcomes were successful. The remaining images were preprocessed again manually, in order to make best use of the available data. Figure 8 shows an example of a TDI image along with its corresponding function (in red) and the beginning and end points of the cycles (with white crosses). Figure 9 shows the two resulting cycles (the first one in solid line, the second one in dotted line) in the same graph. Note that they have been standardized to have the same length. For this image, the selected cycle was the first one, although both of them are quite similar.

Despite the fact that low quality images were not considered in this study, the proposed algorithm still performs well when a portion of the image has a weak signal but contains one or more cycles with an acceptable quality.

## Usage Notes

The preprocessing procedure was implemented in R (version 4.2.2). To reproduce the analyses, the installation of some R packages is required: png^13^, grDevices^11^, raster^14^, rasterVis^15^, lattice^16^ and pracma^17^.

The code can be easily adapted to other type of images. In our case, png images are read in R and the HSV colour of each pixel is considered. Then, the pixels of interest can be selected by taking into account their colours. For TDI images, since the goal are yellow pixels, only the H component is controlled. As the result contains some almost black isolated pixels, a denoising process was performed to get rid of them, by taking into account their own V component and the one of their neighbours. The threshold considered for V can be easily changed to better fit other images, if needed. Regarding the transformation of pixel positions into velocities, it is necessary to know, at least, the velocity that corresponds to two pixels in the image. This way, one can find the conversion factor between pixels and velocities. All this information can be easily changed in the code.

## Data Availability

The commented R code for the preprocessing of image data is available at Figshare^10^. In addition, for reproducibility issues, two TDI images are provided. One of them requires a manual selection of the beginning and end points of the cycle.
